# Deep brain stimulation in the subthalamic nuclei alters postural alignment and adaptation in Parkinson’s disease

**DOI:** 10.1371/journal.pone.0259862

**Published:** 2021-12-14

**Authors:** Per-Anders Fransson, Maria H. Nilsson, Stig Rehncrona, Fredrik Tjernström, Måns Magnusson, Rolf Johansson, Mitesh Patel

**Affiliations:** 1 Department of Clinical Sciences, Lund University, Lund, Sweden; 2 Department of Health Sciences, Lund University, Lund, Sweden; 3 Memory Clinic, Skåne University Hospital, Malmö, Sweden; 4 Clinical Memory Research Unit, Faculty of Medicine, Lund University, Lund, Sweden; 5 Department of Neurosurgery, Lund University, Lund, Sweden; 6 Department of Automatic Control, Lund University, Lund, Sweden; 7 School of Medicine & Clinical Practice, Faculty of Science, University of Wolverhampton, Wolverhampton, United Kingdom; Tokai University, JAPAN

## Abstract

Parkinson’s disease (PD) can produce postural abnormalities of the standing body position such as kyphosis. We investigated the effects of PD, deep brain stimulation (DBS) in the subthalamic nucleus (STN), vision and adaptation on body position in a well-defined group of patients with PD in quiet standing and during balance perturbations. Ten patients with PD and 25 young and 17 old control participants were recruited. Body position was measured with 3D motion tracking of the ankle, knee, hip, shoulder and head. By taking the ankle as reference, we mapped the position of the joints during quiet standing and balance perturbations through repeated calf muscle vibration. We did this to explore the effect of PD, DBS in the STN, and vision on the motor learning process of adaptation in response to the repeated stimulus. We found that patients with PD adopt a different body position with DBS ON vs. DBS OFF, to young and old controls, and with eyes open vs. eyes closed. There was an altered body position in PD with greater flexion of the head, shoulder and knee (p≤0.042) and a posterior position of the hip with DBS OFF (p≤0.014). With DBS ON, body position was brought more in line with the position taken by control participants but there was still evidence of greater flexion at the head, shoulder and knee. The amplitude of movement during the vibration period decreased in controls at all measured sites with eyes open and closed (except at the head in old controls with eyes open) showing adaptation which contrasted the weaker adaptive responses in patients with PD. Our findings suggest that alterations of posture and greater forward leaning with repeated calf vibration, are independent from reduced movement amplitude changes. DBS in the STN can significantly improve body position in PD although the effects are not completely reversed. Patients with PD maintain adaptive capabilities by leaning further forward and reducing movement amplitude despite their kyphotic posture.

## Introduction

Postural abnormalities, such as kyphosis, are common in patients with Parkinson’s disease (PD) and increase in severity with disease progression. One reason for this is that dysfunction of the basal ganglia in PD can lead to alterations of resting body position, muscle tone and inhibition of the latissimus dorsi muscles [1]. The resulting trunk flexion, which is an anterior leaning of the trunk towards the hip during standing [2], increases postural instability and alters walking ability [3]. Medical intervention includes botulinum toxin injections into axial muscles, which is only partially effective. Levodopa or other dopamine agonists can increase the degree of trunk flexion. Deep brain stimulation (DBS) in the subthalamic nucleus (STN) can reduce the degree of trunk flexion [4–6] although a recent meta-analysis [7] has indicated variable effects. Further analysis appears to suggest that the effectiveness of DBS in the STN on trunk flexion is age dependent [8]. Given the potential impact of DBS in the STN on postural abnormalities in PD, we have conducted a double-blind randomized study to investigate the effectiveness of DBS in the STN on body position in quiet upright stance and from balance perturbations.

Maintaining an upright position during stance (postural control) is dependent upon accurate sensory cues. In PD however there is impaired integration of proprioception [9, 10] which may alter the perception of an abnormal trunk position. To compensate for this, postural control in PD becomes increasingly dependent upon trunk muscle strength [11] and visual information [12]. The latter reflects increased visual dependency, which involves a shift to more reliable sensory cues, i.e., to visual afferents. Perturbing stance through calf muscle vibration is one way to further explore postural control, and specifically the contribution of signals from visual afferents [13]. Calf muscle vibration produces an illusion of muscle stretch that results in a posterior movement to correct the perceived imbalance. Furthermore, when repeated, calf muscle vibration results in a reduction of vibration-induced movement through adaptive mechanisms [13–16]. Postural adaptation is a branch of motor learning that is concerned with the modification of motor plans. In PD, motor learning has been the subject of interest due to the implications on balance re-training. However, in an investigation of adaptation using calf muscle vibration, patients with PD had poorer postural control as compared to controls and adaptive responses were almost abolished [17]. Whether this abolition of adaptation extends to whole body position is unclear and the degree to which DBS in the STN alters flexion in terms of whole body position requires further objective studies.

Trunk position in PD has been measured or assumed from muscle echo intensity of the abdominal wall [18], from review of video recordings [4, 19], subjectively using the 0–4 MDS Unified Parkinson’s Disease Rating Scale [20–22], or through observation [23]. Here, we use objective ultrasound motion detection to study and quantify alterations of body position in patients with PD and consider the longer-term effectiveness of DBS in the STN.

This study aimed to explore: 1. The differences between patients with PD and healthy adults on body position and movement amplitude; 2. The effects of DBS in the STN on body position and movement amplitudes in quiet stance and during balance perturbations in patients with PD; 3. The effects of adaptation on body position and movement amplitudes in patients with PD, with DBS ON and OFF, and healthy adults. In all three aims, the effects of vision were also studied by comparing measurements between eyes closed and eyes open. Important aspects to this study were the overnight withdrawal of anti-PD medication and double-blind, randomized, design.

## Materials and methods

### Participants

Fifty-two consenting participants were recruited across three groups: a Parkinson’s disease group (PD) with deep brain stimulation (DBS) electrodes implanted in the subthalamic nucleus (STN); a control group of young adults; and a control group of older adults. The study followed the most recent Declaration of Helsinki and was approved by the Ethical Review Board (411/2006), Lund, Sweden. Oral consent was obtained from all participants before they were included in the study.

The PD group were recruited from 25 eligible patients who fulfilled the inclusion criteria of idiopathic PD responsive to dopamine replacement therapy. Participants were required to be between 50–70 years of age and had bilateral STN stimulation for at least one year. Fifteen patients with PD either declined to participate or met the following exclusion criteria: secondary disorder affecting postural control, suffered from pain or was unable to cooperate. The final PD group had 10 adults (9 men and 1 woman) aged between 59 and 69 years (mean age 64.3 years, SEM 1.3 years; mean height 1.77m, SEM 0.02m; and mean weight 79.6 kg, SEM 2.7kg). The clinical details, such as settings of the DBS devices, medication, UPDRS scores and description of the neurosurgical procedures can be found in Table 1 and in previous articles [17, 24–26].

**Table 1:** Characteristics of Parkinson’s disease patients.

Characteristics of PD patients			Median (range)
Age (years)			66 (59–69)
Gender			9 men, 1 woman
Disease duration (years)			18 (10–22)
Medication as L-dopa equivalent dose (mg/day) ^a^			416 (294–989)
DBS treatment duration (months)			37 (15–70)
DBS pulse settings	Right	Amplitude (V)	3.3 (2.5–4.3)
		Pulse width (μs),	60 (60–90)
		Frequency (Hz)	145 (100–185)
	Left	Amplitude (V)	3.4 (2.2–4.3)
		Pulse width (μs),	60 (60–90)
		Frequency (Hz)	130 (100–185)
Positions of negative polarity contacts with reference to the intercommissural line midpoint	Right (mm)	Lateral	11.7 (10.4–13.1)
		Posterior	3.4 (3.0–4.0)
		Inferior	2.1 (1.0–5.6)
	Left (mm)	Lateral	11.4 (9.6–13.0)
		Posterior	3.5 (3.3–5.2)
		Inferior	2.6 (1.2–4.2)
	Intercommissural line (mm)		24.8 (23.5–25.6)
UPDRS part III scores in anti-PD medication OFF state ^b^			
DBS OFF		Item 20 and 21 (tremor)	2.3 (0–8.1)
		Total Score	41.0 (35.0–83.5)
DBS ON		Item 20 and 21 (tremor)	0 (0–0)
		Total Score	21.5 (11.0–30.5)

^a^ Calculated equivalent doses of Levodopa according to the method presented by Østergaard et al. [27], and Calne [28]. All participants received L-dopa in their daily life and 7/10 subjects received also dopamine agonists.

^b^ UPDRS part III: Unified Parkinson’s disease Rating Scale, motor examination. The maximum total score is 108 points, where higher scores reflect more severe motor symptoms. The evaluations were performed in anti-PD medication OFF state. All anti-Parkinsonian medications were withdrawn overnight for 10–12 hours. The UPDRS assessments were done at the same occasion as the assessments of posture and body movement amplitudes.

The “young” control group comprised 25 healthy adults (12 men and 13 women) aged 19 to 41 years (mean age 25.1 years, SEM 0.9 years; mean height 1.75m, SEM 0.02m; and mean weight 68.8 kg, SEM 2.7kg). The “old” control group comprised 17 healthy adults (9 men and 8 women) aged 65 to 79 years (mean 71.2 years, SEM 1.0 years; mean weight 80.1kg, SEM 2.9kg; and mean height 1.67m, SEM 0.02m). Health checks were performed by physicians to ensure their healthy status, and ruling out individuals who had experienced unexplained falls or neurological/musculoskeletal conditions. Participants were instructed to avoid alcohol at least 48 hours before testing.

### Equipment

An ultrasound 3D-Motion Analysis system (Zebris^™^ CMS-HS) was used for recording the movements of five anatomical bony landmarks, using position marked placed on the right side of the subject’s body. The marker denoted “Head” was attached to the *os zygomaticum*; the marker denoted “Shoulder” to the *tuberculum majus*; the marker denoted “Hip” to the *crista iliaca*; the marked denoted “Knee” to the *lateral epicondyle of femur*, and the marker denoted “Ankle” to the *lateral distal fibula head*. The marker movements in 3D space were recorded at 50 Hz with about 0.4 mm precision.

### Procedure

The PD group were kept as in-patients the night before testing and all anti-PD medications were withdrawn. Medication was withdrawn from 10pm and testing began the following morning at 9am. DBS was programmed to deliver STN stimulation (ON) or not to deliver STN stimulation (OFF) at least 30mins before testing. The DBS setting was programmed and concealed to ensure a double-blind design by an independent health care professional. The ON/OFF settings of the DBS and the order in which posturography was performed with eyes closed (EC) and eyes open (EO), were randomized using a Latin Square design, to avoid systematic test order bias. Another reason for a counter-balanced test order design was to minimize systematic order effects from the withdrawal of medication and from the changes in DBS ON/OFF status. A change in DBS status and a withdrawal of anti-PD medications may reach its full effect at different times across a PD population. Therefore, no tests were performed within 30 minutes from DBS programming.

In both control groups, the test order of performing posturography with EC or EO first was randomized using a Latin square design, thus matching the procedures applied in the PD group. The posturography test had an initial 35-seconds of quiet stance, followed by 200-seconds of randomized balance perturbations. Randomized balance perturbations were generated by vibrators placed over the gastrocnemius muscles of both legs. The vibrators were 6cm long and 1cm in diameter and standardized to create a vibration of 1.0mm amplitude and 85Hz frequency. The vibrations were applied as sequences of ON/OFF pulses, with durations between 0.8 to 6.4 seconds. To ensure a standardized stimulation sequence a pseudorandom binary sequence (PRBS) schedule was used [29, 30]. All participants were submitted to the same stimulation sequence, and the sequence was identical during tests with eyes closed and eyes open.

Before starting the posturography test, participants were asked to fold their arms across the chest and stand in a comfortable erect and relaxed posture. Participants stood barefoot on a flat, hard surface. Guidelines on the floor were used to position the heels 3cm apart and the feet at an angle of 30° open to the front. Participants stood 1.5m in front of a wall and when the tests were performed with eyes open the participants were instructed to focus on an image (6cm x 4cm large) placed at eye level on the wall. Participants were allowed to rest for 5 minutes between the two tests. During both posturography tests, the participants listened to calm classical music through headphones to reduce movement references and avoid extraneous sound distractions [31]. All participants were naïve to the vibratory stimulus and its effects on balance.

### Analysis

As calf muscle vibration primarily induces body movement in an anteroposterior direction, only movement in this direction was analyzed [30, 32]. The posture alignment and movement amplitudes were determined by calculating the anteroposterior angular positions of the ‘Head’, ‘Shoulder’, ‘Hip’ and ’Knee’ markers during quiet stance (0–30s) and during four consecutive 50-second periods during the balance perturbation phase (period 1: 30–80s; period 2: 80–130s; period 3: 130–180s; period 4: 180–230s). On sample level, the angular anteroposterior position of each individual marker was calculated from the marker’s height and anteroposterior position using the ankle marker as the stable relative zero-position reference [13, 33]. For each marker and for the 5 periods analyzed, the posture alignment was obtained by calculating the median angular position (50% percentile) of all angular movements recorded during the period. The ranges forwards and backwards were calculated from the 5% (most forward) and 95% (most backward) percentile values. The angular movement amplitudes were calculated by subtracting the 5% (most forward) and 95% (most backward) percentile values from each other [33].

### Statistical analysis

The anteroposterior posture alignment and the movement amplitudes recorded for the body sites ‘Head’, ‘Shoulder’, ‘Hip’ and ’ Knee’ during quiet stance and the four 50-s periods of balance perturbations were analyzed with a repeated measures General Linear Model analysis of variance (GLM ANOVA). The analyses determined the role of DBS in the PD participant category, verified differences between group categories, as well as determined the effects of visual cues and the adaptive strategy. GLM ANOVA were performed after a validation of the appropriateness of the statistical method given the properties of the data sets and model residuals [34]. The main factor combinations analyzed for their effects on body posture and of the amplitudes of the body movements made at each site during balance perturbations were:

1. DBS (OFF vs ON, df 1), Vision (EO vs. EC, df 1) and Adaptation (Periods 1–4, df 3).
2. Group (PD OFF vs Old controls), Vision (EO vs. EC) and Adaptation (Periods 1–4).
3. Group (PD ON vs Old controls), Vision (EO vs. EC) and Adaptation (Periods 1–4).
4. Group (PD OFF vs Young controls), Vision (EO vs. EC) and Adaptation (Periods 1–4).
5. Group (PD ON vs Young controls), Vision (EO vs. EC) and Adaptation (Periods 1–4).
6. Group (Young vs Old controls), Vision (EO vs. EC) and Adaptation (Periods 1–4).

In analysis 1) the model parameter DBS is a Within-Subjects variable. In analyses 2–6), the model parameter Group is a Between-Subjects factor. In analyses 1–6), the model parameters Vision and Adaptation are Within-Subjects variables.

In the post-hoc analyses, Wilcoxon within-subject comparisons were performed to determine whether the quiet stance posture and the amplitudes of the body movements made changed as compared to during period 1 with balance perturbations. Moreover, the accumulated changes made during the balance perturbation phase was determined by comparing the posture and the amplitudes of the body movements made during period 1 and during period 4. Procedures were utilized to address potential Type I and Type II errors. Thus, the significant p-value level was set to p<0.025 in the post hoc tests, adhering to the Bonferroni correction method. The significant p-value level was set to p<0.05 in the repeated measures GLM ANOVA analyses. Trends < 0.1 are marked in the tables. As not all datasets were normally distributed before or after logarithmic transformation, non-parametric statistics were used in the statistical evaluation.

## Results

### Angular position (body posture)

#### Effects of vision and adaptation on body posture during balance perturbations

**Fig 1:**
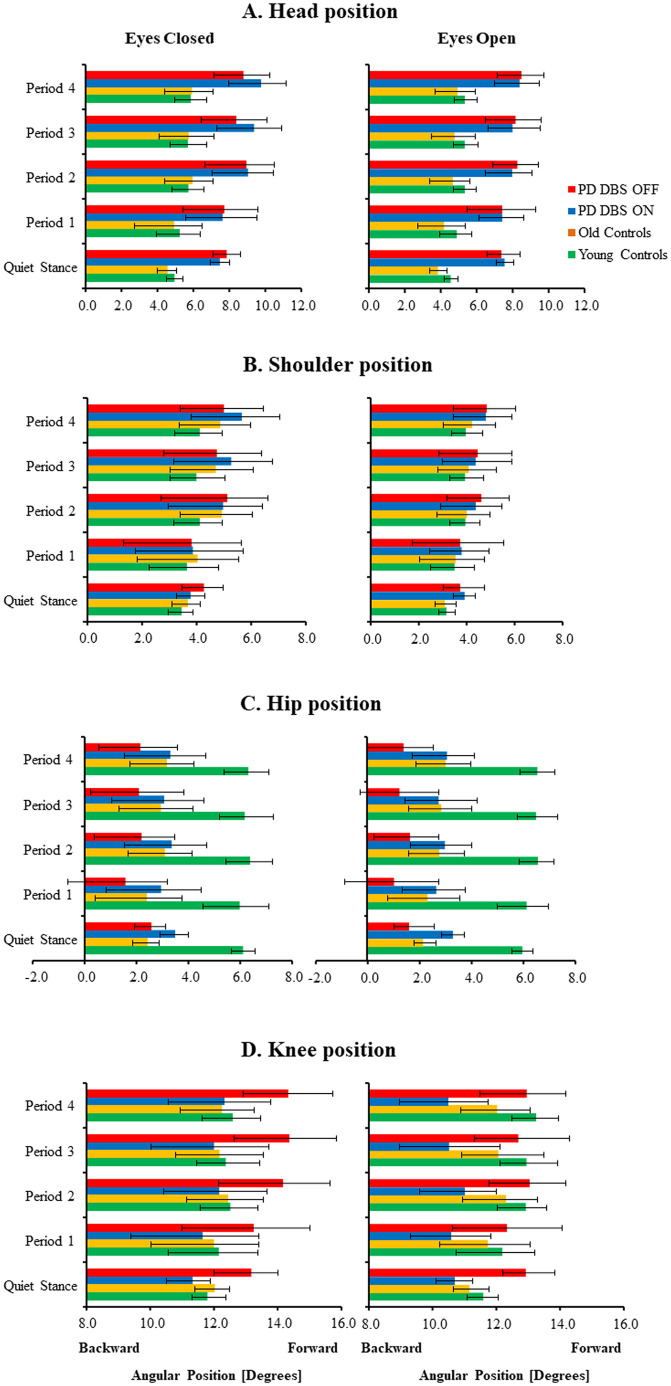
Body posture during different phases of the posturography tests. Results are presented for tests with eyes closed and eyes open in patients with PD with DBS OFF; in patients with PD with DBS ON; in old and in young controls. Fig 1A present the head position, Fig 1B the shoulder position, Fig 1C the hip position and Fig 1D the knee position. The values are in degrees, where the size of the bars represent group mean values of the posture and the whiskers mark respectively group mean values of range forward (increasing positive) and range backward (decreasing positive). All subjects utilize an increasing body leaning forward during balance perturbations, but this change was effectuated stronger in both control groups.

**Fig 2:**
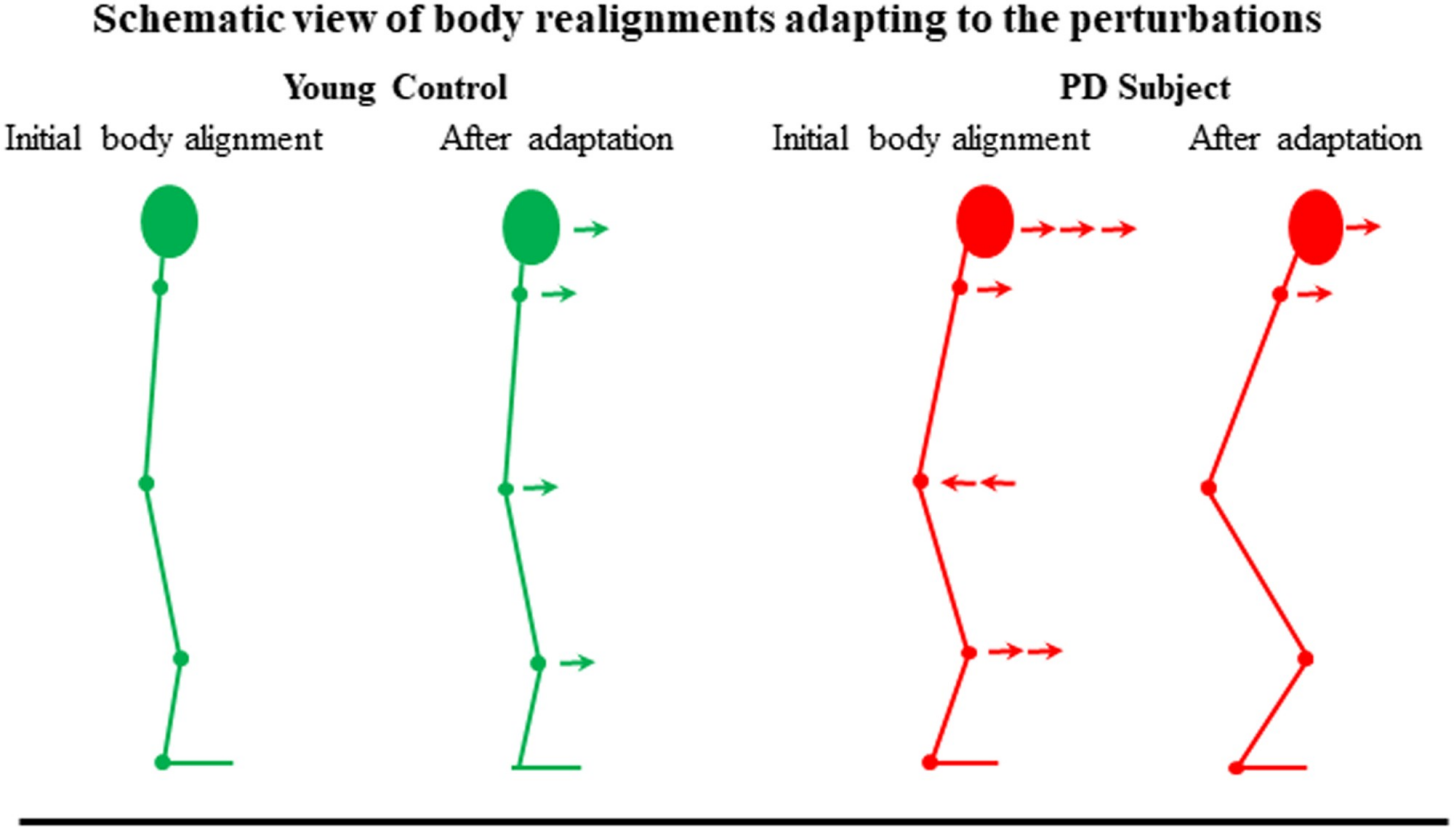
Schematic image of the changes of posture. The figure illustrates the typical posture of young controls and of patients with PD in DBS OFF (both with eyes closed and eyes open). During balance perturbations, young controls increased their leaning forward involving all body segments. In patients with PD with DBS OFF, the posture was deformed as compared to young controls by an increased knee bending, that the hip was more backward and that the head was particularly positioned more forward. During balance perturbations, the PD subjects increased the leaning forward of the shoulders and head.

Fig 1 shows the angular positions and movement ranges at the head, shoulder, hip and knee for the three different groups. The most notable differences between categories were for the positions of the head and hip. In all group categories, body leaning increased to a more forward position over time during the vibration period. As illustrated schematically in Fig 2, patients with PD tended to flex the knees more, position the hips further backward and position the head further forward than young controls. During vibration, young controls increased body leaning at all recorded sites with adaptation, whereas in PD, the head and shoulders were mostly further forward.

#### Effects of DBS, vision and adaptation

**Fig 3:**
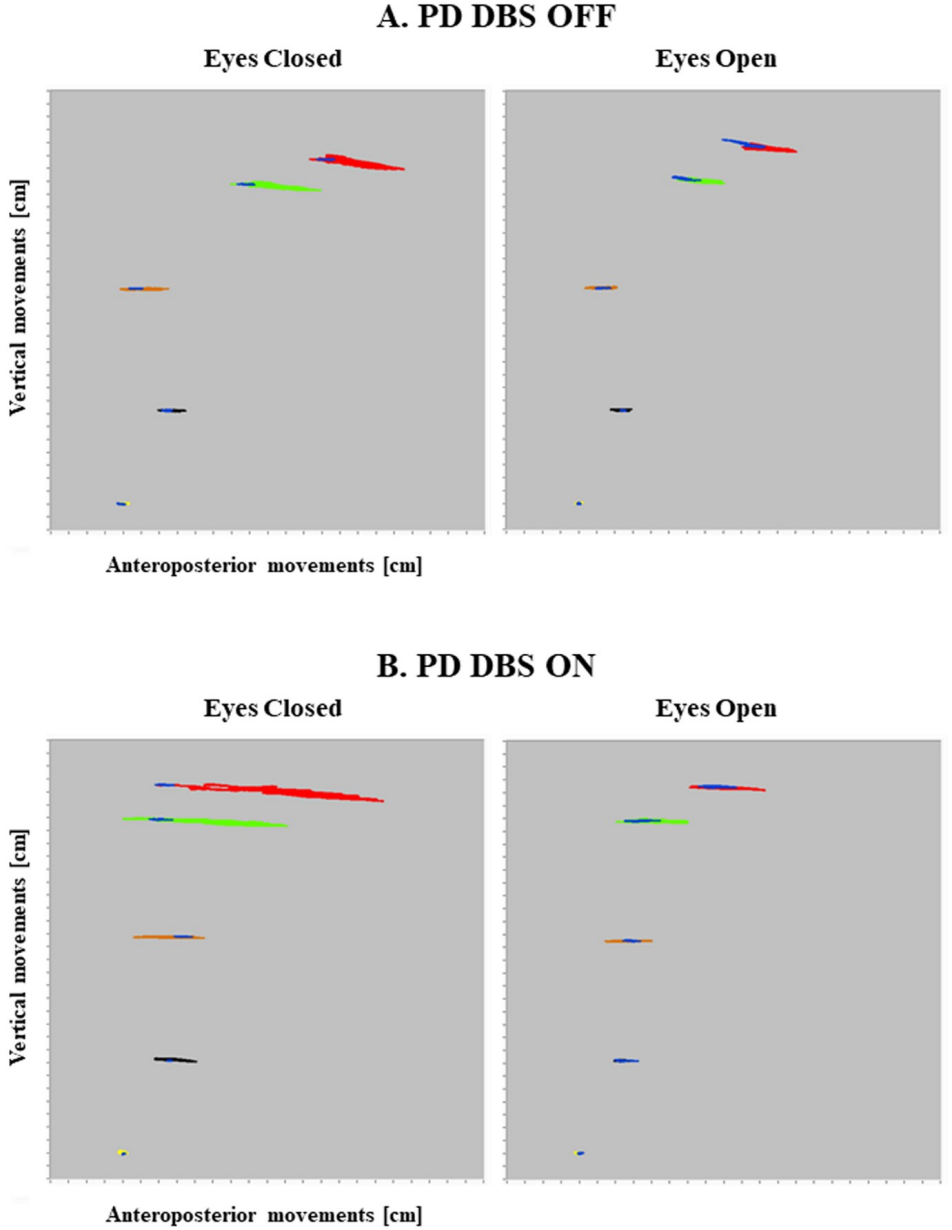
Body movement recordings from a PD subject during DBS OFF and DBS ON. The figure presents the body movements during Fig 3A: DBS OFF and Fig 3B: DBS ON. The red color marks the head movement; the green color the shoulder movement; the orange color the hip movement; the black color the knee movement and the yellow color the ankle movement. The blue segment at each marker position marks the movements made during quiet stance. The sizes of all body movements were reduced in DBS OFF as compared to with DBS ON. However, the posture was deformed with DBS OFF by increased leaning forward of the head, shoulder and knees and by a more backward position of the hip.

Fig 3 shows the angular movements in one participant with PD during DBS OFF and DBS ON. Through adaptation, there was a reduced amplitude of movement but the body position remained fairly fixed in relation to the position of the ankle. However, with DBS OFF, body position was altered with an increased forward leaning, specifically the position of the head, shoulders and knees were further forward in relation to the ankle, and by a more posterior positioning of the hip, compared to DBS ON.

From the PD group data, the GLM ANOVA of the main factor, DBS, revealed that the knees were further flexed with DBS OFF (p = 0.030, see Table 2 and Fig 1). The main factor, vision, revealed that the knees were further flexed with eyes closed than with eyes open (p = 0.032). The main factor, adaptation, revealed that the patients with PD made marked changes over time of the body positions at all recorded sites to a more forward leaning (i.e., the knee, hip, shoulder and head were further forward in relation to the ankle) (p≤0.014).

**Table 2:** DBS, vision and adaptation effects on the body posture.

GLM ANOVA Statistics ^a^	DBS	Vision	Adaptation	Group x Vision	Group x Adaptation	Vision x Adaptation	Group x Vision x Adaptation
**DBS OFF vs ON**							
Head	0.580 (0.3)	*0*.*095 (3*.*9)*	**<0.001 (50.4)**	0.246 (1.7)	0.559 (0.4)	**0.021 (9.7)**	0.678 (0.2)
Shoulder	0.661 (0.2)	0.376 (0.9)	**<0.001 (56.9)**	0.373 (0.9)	0.272 (1.5)	**0.014 (11.6)**	0.604 (0.3)
Hip	0.175 (2.4)	0.705 (0.2)	**<0.001 (47.1)**	0.266 (1.5)	0.283 (1.4)	0.312 (1.2)	0.360 (1.0)
Knee	**0.030 (8.1)**	**0.032 (7.8)**	**0.014 (11.8)**	0.817 (0.1)	0.236 (1.7)	*0*.*050 (6*.*0)*	0.141 (2.9)

^a^ Repeated measures GLM ANOVA of the body posture with main factors “DBS”, “Vision” and “Adaptation” and their factor interactions. The F-values are presented within the parenthesis. The significant differences are marked in bold text and the trends in italic text.

The significant interaction between Vision x Adaptation for the head (p = 0.021) and for the shoulder (p = 0.014) shows increased forward leaning with eyes closed than with eyes open.

#### Effects of DBS, vision and adaptation in PD vs old controls comparisons

The GLM ANOVA of the main factor, group, revealed that the head was positioned further forward with DBS OFF (p = 0.020) and DBS ON (p<0.001) as compared to old controls (Table 3 and Fig 1). The main factor, vision, revealed that the head was positioned further forward with eyes closed than with eyes open (p = 0.005) with DBS OFF. Moreover, the head and shoulders were positioned further forward with eyes closed than with eyes open (p≤0.015) with DBS ON. The main factor, adaptation, revealed that the patients with PD and old controls made marked changes over time of the body positions at all recorded sites to a more forward leaning. This occurred in patients with PD with DBS OFF (p<0.001) and DBS ON (p<0.001).

**Table 3:** Group, vision and adaptation effect on the body posture.

GLM ANOVA Statistics ^a^	Group	Vision	Adaptation	Group x Vision	Group x Adaptation	Vision x Adaptation	Group x Vision x Adaptation
**PD OFF vs Old**							
Head	**0.020 (6.3)**	**0.004 (10.1)**	**<0.001 (38.1)**	0.530 (0.4)	0.303 (1.1)	*0*.*050 (4*.*3)*	0.120 (2.6)
Shoulder	0.954 (0.0)	0.218 (1.6)	**<0.001 (43.2)**	0.332 (1.0)	0.157 (2.1)	**0.032 (5.3)**	0.142 (2.3)
Hip	0.491 (0.5)	0.638 (0.2)	**<0.001 (40.8)**	0.436 (0.6)	0.492 (0.5)	0.115 (2.7)	0.393 (0.8)
Knee	0.357 (0.9)	0.166 (2.0)	**<0.001 (21.5)**	0.349 (0.9)	0.653 (0.2)	0.402 (0.7)	0.116 (2.7)
**PD ON vs Old**							
Head	**<0.001 (15.4)**	**<0.001 (17.1)**	**<0.001 (46.3)**	0.965 (0.0)	*0*.*067 (3*.*7)*	**0.007 (8.8)**	0.119 (2.6)
Shoulder	0.631 (0.2)	**0.015 (6.8)**	**<0.001 (53.9)**	0.906 (0.0)	**0.025 (5.7)**	**0.009 (8.0)**	0.100 (2.9)
Hip	0.672 (0.2)	0.391 (0.8)	**<0.001 (60.6)**	0.574 (0.3)	0.169 (2.0)	0.354 (0.9)	0.354 (0.9)
Knee	0.451 (0.6)	0.172 (2.0)	**<0.001 (28.7)**	0.358 (0.9)	0.324 (1.0)	0.676 (0.2)	0.213 (1.6)
**PD OFF vs Young**							
Head	**0.007 (8.3)**	**0.035 (4.9)**	**<0.001 (40.7)**	0.556 (0.4)	**0.028 (5.4)**	**0.006 (8.6)**	**0.031 (5.1)**
Shoulder	0.621 (0.2)	0.692 (0.2)	**<0.001 (47.0)**	0.930 (0.0)	**0.011 (7.3)**	*0*.*067 (3*.*6)*	**0.046 (4.3)**
Hip	**<0.001 (46.2)**	0.234 (1.5)	**<0.001 (41.6)**	0.899 (0.0)	0.245 (1.4)	0.404 (0.7)	0.127 (2.5)
Knee	0.355 (0.9)	0.317 (1.0)	**<0.001 (24.7)**	**0.017 (6.4)**	0.500 (0.5)	0.227 (1.5)	**0.005 (9.2)**
**PD ON vs Young**							
Head	**<0.001 (21.6)**	**0.003 (10.1)**	**<0.001 (53.1)**	0.173 (1.9)	**<0.001 (13.8)**	**0.013 (6.9)**	**0.010 (7.5)**
Shoulder	0.249 (1.4)	0.100 (2.9)	**<0.001 (62.5)**	0.278 (1.2)	**<0.001 (17.5)**	**0.048 (4.2)**	**0.033 (5.0)**
Hip	**<0.001 (37.7)**	0.800 (0.1)	**<0.001 (61.0)**	0.187 (1.8)	0.165 (2.0)	0.372 (0.8)	0.398 (0.7)
Knee	0.132 (2.4)	0.359 (0.9)	**<0.001 (33.9)**	**0.025 (5.5)**	**0.042 (4.5)**	0.165 (2.0)	**0.025 (5.6)**
**Old vs. Young**							
Head	0.397 (0.7)	**<0.001 (23.7)**	**<0.001 (60.8)**	**0.036 (4.7)**	**0.043 (4.4)**	**0.002 (10.5)**	**0.030 (5.1)**
Shoulder	0.319 (1.0)	**0.014 (6.7)**	**<0.001 (66.4)**	*0*.*088 (3*.*1)*	*0*.*095 (2*.*9)*	**0.001 (12.1)**	*0*.*075 (3*.*3)*
Hip	**<0.001 (62.0)**	0.595 (0.3)	**<0.001 (77.1)**	0.317 (1.0)	*0*.*097 (2*.*9)*	**0.040 (4.5)**	0.249 (1.4)
Knee	0.627 (0.2)	0.682 (0.2)	**<0.001 (48.8)**	0.264 (1.3)	0.167 (2.0)	**0.029 (5.1)**	*0*.*062 (3*.*7)*

^a^ Repeated measures GLM ANOVA of the body posture with main factors “Group”, “Vision” and “Adaptation” and their factor interactions. The F-values are presented within the parenthesis. The significant differences are marked in bold text and the trends in italic text.

The significant interaction between Group x Adaptation revealed that the shoulders were positioned further forward (p = 0.025) with DBS ON compared to old controls. The significant interaction between Vision x Adaptation revealed that the shoulders were positioned further forward with eyes closed than with eyes open (p = 0.005) with DBS OFF. Moreover, the head and shoulders (p≤0.009) were positioned further forward with eyes closed than with eyes open with DBS ON.

#### Effects of DBS, vision and adaptation in PD vs younger controls comparisons

The GLM ANOVA of the main factor, group, revealed that the head and hip were positioned further forward in DBS OFF (p≤0.007) and DBS ON (p<0.001) compared to young controls (Table 3 and Fig 1). The main factor, vision, revealed that the head was positioned further forward with eyes closed than with eyes open with DBS OFF (p = 0.035) and DBS ON (p = 0.003). The main factor, adaptation, revealed that in patients with PD and young controls body positions were further forward over time during the vibration period. This occurred in patients with PD with DBS OFF (p<0.001) and DBS ON (p<0.001).

The significant interaction between Group x Vision revealed that the knees were flexed further with DBS OFF (p = 0.017) and DBS ON (p = 0.025) with eyes closed compared to all other conditions and young controls. The significant interaction between Group x Adaptation revealed that the head and shoulders (p≤0.028) moved further forward over time with DBS OFF compared to young controls. Moreover, the head, shoulders and knees (p≤0.042) moved further forward with DBS ON compared to young controls. The significant interaction between Vision x Adaptation revealed that the head moved further forward with eyes closed than with eyes open (p = 0.006) with DBS OFF. Moreover, the head and shoulders (p≤0.048) were positioned further forward with eyes closed than with eyes open with DBS ON.

Finally, the significant interaction between Group x Vision x Adaptation revealed that the head, shoulders and knees moved forwards faster (i.e., in earlier vibration periods) with eyes closed with DBS OFF (p≤0.046) and DBS ON (p≤0.033) compared to young controls.

#### Effects of vision and adaptation in old vs young controls comparisons

GLM ANOVA of the main factor, group, revealed that the hip was positioned further forward in young controls compared to old controls (p<0.001) (Table 3 and Fig 1). The main factor, vision, revealed that the head and shoulders (p≤0.014) were positioned further forward with eyes closed than with eyes open. The main factor, adaptation, revealed that the young and old controls moved the head, shoulder, hip and knee positions further forward over time (i.e., increased leaning) (p<0.001).

The significant interaction between Group x Vision revealed that the head was further forward (p = 0.036) with eyes closed in the young control compared to old controls. The significant interaction between Group x Adaptation revealed that the head increasingly moved further forward (p = 0.043) in old controls compared to young controls. The significant interaction between Vision x Adaptation revealed that the head, shoulders and hip (p≤0.040) positions were positioned further forward with eyes closed than with eyes open. However, the knees (p = 0.029) were flexed further forward with eyes open than with eyes closed.

Finally, the significant interaction between Group x Vision x Adaptation revealed that the head (p = 0.030) was positioned less forward over time in old controls with eyes open compared to young controls.

### Body position changes to initial and continuous balance perturbations

#### PD with DBS OFF

With DBS OFF, post hoc analyses revealed that the PD group moved the hip further back (p = 0.020) during period 1 from quiet stance with eyes open (Table 4 and Fig 1). During vibration, from period 1 to period 4, the head moved further forward (p = 0.016) with eyes closed, and the shoulders further forward (p = 0.006) with eyes open.

**Table 4:** Body posture changes between quiet stance and vibration period 1 and between vibration period 1 and period 4.

Body posture changes ^a^	Quiet stance vs Vibration Period 1		Vibration Period 1 vs Period 4	
	Eyes Closed	Eyes Open	Eyes Closed	Eyes Open
**PD OFF**				
Head	0.734 (0.98)	0.625 (1.01)	**0.016 (1.14)**	*0*.*027 (1*.*15)*
Shoulder	0.250 (0.90)	0.770 (1.00)	*0*.*047 (1*.*31)*	**0.006 (1.30)**
Hip	*0*.*039 (0*.*61)*	**0.020 (0.64)**	0.219 (1.36)	0.232 (1.36)
Knee	0.910 (1.01)	*0*.*064 (0*.*95)*	0.219 (1.08)	0.322 (1.05)
**PD ON**				
Head	0.695 (1.02)	0.770 (0.98)	**0.002 (1.28)**	**0.002 (1.13)**
Shoulder	0.922 (1.02)	0.625 (0.97)	**0.002 (1.47)**	**0.002 (1.27)**
Hip	**0.010 (0.84)**	**0.014 (0.80)**	0.275 (1.12)	0.232 (1.16)
Knee	0.432 (1.03)	0.203 (0.99)	0.557 (1.06)	0.820 (0.99)
**Old Controls**				
Head	0.109 (1.08)	**0.018 (1.09)**	**0.006 (1.20)**	**<0.001 (1.18)**
Shoulder	0.123 (1.10)	**0.013 (1.14)**	**0.005 (1.21)**	**<0.001 (1.20)**
Hip	0.890 (0.99)	**0.013 (1.08)**	**<0.001 (1.32)**	**0.001 (1.30)**
Knee	0.517 (1.00)	*0*.*027 (1*.*05)*	0.611 (1.02)	0.431 (1.03)
**Young Controls**				
Head	*0*.*036 (1*.*06)*	*0*.*045 (1*.*07)*	**<0.001 (1.12)**	**<0.001 (1.09)**
Shoulder	0.410 (1.06)	*0*.*053 (1*.*11)*	**0.003 (1.13)**	**<0.001 (1.13)**
Hip	0.229 (0.98)	0.513 (1.03)	**0.005 (1.06)**	**0.010 (1.07)**
Knee	0.190 (1.03)	*0*.*039 (1*.*05)*	**0.012 (1.04)**	**<0.001 (1.09)**

^a^ The mean quotient value between quiet stance and period 1 and between period 1 and period 4 are presented within the parenthesis. A quotient value above 1.0 signifies an increased body leaning forward from the first to the second time period. The significant differences are marked in bold text and the trends in italic text.

#### PD with DBS ON

With DBS ON, post hoc analyses revealed that the PD group moved the hip further back both with eyes closed (p = 0.010) and with eyes open (p = 0.014) from quiet stance to period 1 (Table 4 and Fig 1). During the vibration period, from period 1 to period 4, the head and shoulders moved further forward with eyes closed (p = 0.002), and the head and shoulders moved more forward with eyes open (p = 0.002).

#### Old controls

In old controls, post hoc analyses revealed that from quiet stance to period 1, all body positions except the knee were further forwards (p≤0.018) with eyes open (Table 4 and Fig 1). During the vibration period, from period 1 to period 4, the head, shoulders and hip moved further forward (p≤0.006) with eyes closed, and the head, shoulders and hip moved further forward (p≤0.001) with eyes open.

#### Young controls

In young controls, post-hoc analyses revealed that from quiet stance to period 1, no significant changes of posture were made (Table 4 and Fig 1). During the vibration period, from period 1 to period 4, all body positions were further forward with eyes closed (p≤0.012) and with eyes open (p≤0.010).

### Body movement amplitude

#### Effects of vision and adaptation on body movement amplitude during balance perturbations

**Fig 4:**
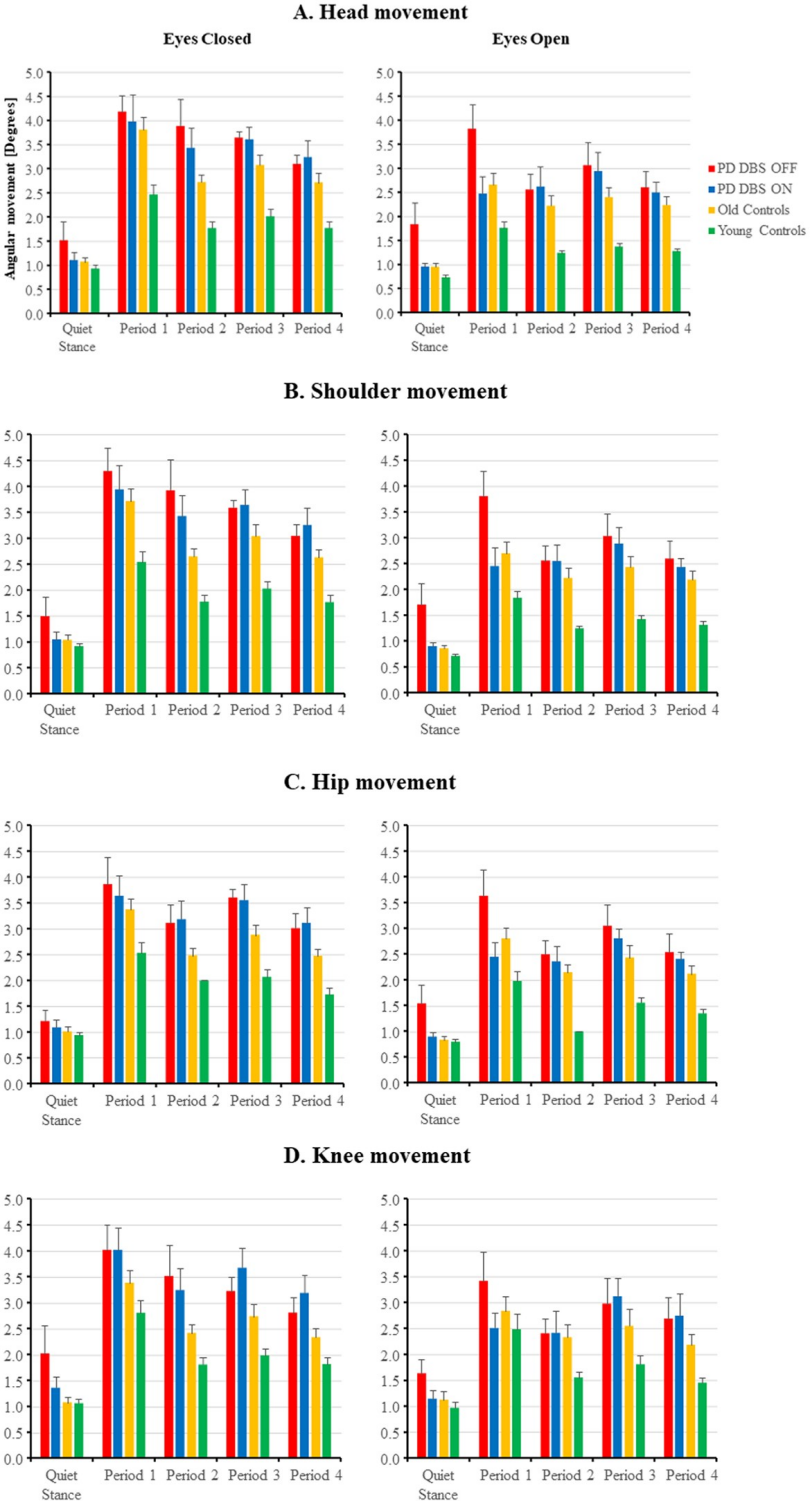
Body movement sizes during different phases of the posturography tests. Results are presented for tests with eyes closed and eyes open in patients with PD with DBS OFF; in patients with PD with DBS ON; in old and in young controls. Fig 4A presents the head movements, Fig 4B the shoulder movements, Fig 4C the hip movements and Fig 4D the knee movements. The values are in degrees, where the size of the bars represent group mean values and the whiskers mark the SEM values. All group categories decreased their body movements at all sites during balance perturbations, signifying a decreasing impact of the balance perturbations.

Fig 4 shows the simultaneously recorded movement amplitudes at the head, shoulder, hip and knee sites for the three different group categories. The amplitudes of the movements made at all recorded sites were clearly smaller in the young controls compared to the PD group. However, the movement amplitudes were not noticeably different between the PD group and the older controls. Moreover, in all group categories, and at all recorded sites, the amplitude of body movement was reduced over time during the vibration period.

#### Effects of DBS, vision and adaptation

The GLM ANOVA of the main factor, vision, revealed that the movement amplitudes were larger with eyes closed than with eyes open at all recorded sites (p≤0.020) (Table 5 and Fig 4). The main factor, adaptation, revealed that the PD group made marked reductions of the body movement over time at all recorded sites (p<0.001).

**Table 5:** DBS, vision and adaptation effects on the size of the body movements.

GLM ANOVA Statistics ^a^	DBS	Vision	Adaptation	Group x Vision	Group x Adaptation	Vision x Adaptation	Group x Vision x Adaptation
**DBS OFF vs ON**							
Head	0.593 (0.3)	**0.020 (9.8)**	**<0.001 (36.0)**	0.779 (0.1)	0.221 (1.9)	0.174 (2.4)	0.341 (1.1)
Shoulder	0.415 (0.8)	**0.012 (12.7)**	**<0.001 (35.6)**	0.860 (0.0)	**0.047 (6.2)**	*0*.*067 (5*.*0)*	0.385 (0.9)
Hip	0.473 (0.6)	**0.007 (15.8)**	**<0.001 (48.9)**	0.914 (0.0)	*0*.*084 (4*.*3)*	0.131 (3.1)	0.594 (0.3)
Knee	0.830 (0.1)	**0.004 (20.9)**	**<0.001 (74.9))**	0.747 (0.1)	0.120 (3.3)	**0.041 (6.8)**	0.273 (1.5)

^a^ Repeated measures GLM ANOVA of the size of the movements with main factors “DBS”, “Vision” and “Adaptation” and their factor interactions. The F-values are presented within the parenthesis. The significant differences are marked in bold text and the trends in italic text.

The significant interaction between Group x Adaptation shows that shoulder movements were reduced proportionally more over time with DBS OFF than with DBS ON (p = 0.047). The significant interaction between Vision x Adaptation shows that knee movements were reduced proportionally more with eyes closed than with eyes open (p = 0.041).

#### Effects of vision and adaptation in PD vs old controls comparisons

The GLM ANOVA of the main factor, vision, revealed that all body movements were larger with eyes closed than with eyes open both with DBS OFF (p<0.001) and with DBS ON (p<0.001) (Table 6 and Fig 4). The main factor, adaptation, revealed that the PD group and old controls made marked reductions over time of body movements at all recorded sites both with DBS OFF (p<0.001) and DBS ON (p<0.001).

**Table 6:** Group, vision and adaptation effect on the size of the body movements.

GLM ANOVA Statistics ^a^	Group	Vision	Adaptation	Group x Vision	Group x Adaptation	Vision x Adaptation	Group x Vision x Adaptation
**PD OFF vs Old**							
Head	0.199 (1.8)	**<0.001 (32.7)**	**<0.001 (43.1)**	0.387 (0.8)	0.378 (0.8)	0.111 (2.8)	0.217 (1.6)
Shoulder	0.150 (2.2)	**<0.001 (34.3)**	**<0.001 (40.2)**	0.197 (1.8)	0.351 (0.9)	*0*.*078 (3*.*4)*	0.305 (1.1)
Hip	0.185 (1.9)	**<0.001 (25.9)**	**<0.001 (73.3)**	0.159 (2.1)	0.563 (0.3)	0.347 (0.9)	0.911 (0.0)
Knee	0.484 (0.5)	**<0.001 (15.9)**	**<0.001 (20.4)**	*0*.*053 (4*.*2)*	0.725 (0.1)	*0*.*075 (3*.*5)*	0.406 (0.7)
**PD ON vs Old**							
Head	0.238 (1.5)	**<0.001 (25.7)**	**<0.001 (27.5)**	0.465 (0.6)	**0.049 (4.3)**	**0.017 (6.5)**	0.483 (0.5)
Shoulder	0.220 (1.6)	**<0.001 (32.1)**	**<0.001 (30.5)**	0.218 (1.6)	**0.037 (4.9)**	**0.015 (6.9)**	0.469 (0.5)
Hip	0.242 (1.4)	**<0.001 (32.0)**	**<0.001 (41.8)**	*0*.*085 (3*.*2)*	*0*.*053 (4*.*1)*	**0.046 (4.4)**	0.527 (0.4)
Knee	0.387 (0.8)	**<0.001 (20.6)**	**<0.001 (29.9)**	**0.016 (6.8)**	*0*.*067 (3*.*7)*	**0.032 (5.2)**	0.329 (1.0)
**PD OFF vs Young**							
Head	**<0.001 (44.6)**	**<0.001 (42.3)**	**<0.001 (77.4)**	0.164 (2.0)	**0.018 (6.3)**	0.233 (1.5)	0.299 (1.1)
Shoulder	**<0.001 (40.1)**	**<0.001 (43.0)**	**<0.001 (80.8)**	*0*.*092 (3*.*0)*	*0*.*098 (2*.*9)*	*0*.*072 (3*.*5)*	0.355 (0.9)
Hip	**<0.001 (21.9)**	**<0.001 (30.0)**	**<0.001 (118.1)**	0.159 (2.1)	**0.041 (4.6)**	0.368 (0.8)	0.705 (0.1)
Knee	**0.003 (10.2)**	**<0.001 (17.4)**	**<0.001 (42.6)**	*0*.*069 (3*.*6)*	0.480 (0.5)	0.247 (1.4)	0.230 (1.5)
**PD ON vs Young**							
Head	**<0.001 (34.7)**	**<0.001 (33.0)**	**<0.001 (40.4)**	0.233 (1.5)	**0.028 (5.3)**	**0.026 (5.5)**	0.139 (2.3)
Shoulder	**<0.001 (33.5)**	**<0.001 (41.2)**	**<0.001 (50.3)**	0.104 (2.8)	**0.021 (5.9)**	**0.009 (7.8)**	*0*.*090 (3*.*1)*
Hip	**<0.001 (25.7)**	**<0.001 (39.1)**	**<0.001 (55.8)**	*0*.*074 (3*.*4)*	**0.044 (4.4)**	**0.035 (4.9)**	0.238 (1.4)
Knee	**<0.001 (14.2)**	**<0.001 (23.8)**	**<0.001 (55.3)**	**0.016 (6.4)**	*0*.*057 (3*.*9)*	0.147 (2.2)	0.159 (2.1)
**Old vs. Young**							
Head	**<0.001 (37.5)**	**<0.001 (57.3)**	**<0.001 (78.1)**	0.613 (0.3)	0.183 (1.8)	**0.025 (5.4)**	0.162 (2.0)
Shoulder	**<0.001 (33.5)**	**<0.001 (50.3)**	**<0.001 (88.7)**	0.776 (0.1)	0.208 (1.6)	**0.028 (5.2)**	0.195 (1.7)
Hip	**<0.001 (22.9)**	**<0.001 (31.1)**	**<0.001 (108.7)**	0.888 (0.0)	0.165 (2.0)	0.180 (1.9)	0.510 (0.4)
Knee	**0.006 (8.4)**	**0.006 (8.5)**	**<0.001 (50.2)**	0.824 (0.1)	0.304 (1.1)	0.115 (2.6)	0.223 (1.5)

^a^ Repeated measures GLM ANOVA of the size of the movements with main factors “Group”, “Vision” and “Adaptation” and their factor interactions. The F-values are presented within the parenthesis. The significant differences are marked in bold text and the trends in italic text.

The significant interaction between Group x Vision revealed that with DBS ON knee movements were larger with eyes closed compared to all other conditions and old controls (p = 0.016). The significant interaction between Group x Adaptation revealed that the head and shoulder (p≤0.037) movements were proportionally reduced with DBS ON less than in old controls. The significant interaction between Vision x Adaptation revealed that all body movements were proportionally reduced more with eyes closed than with eyes open (p≤0.046) in the DBS ON.

#### Effects of DBS, vision and adaptation in PD vs younger controls comparisons

The GLM ANOVA of the main group revealed that all body movements were larger with DBS OFF (p≤0.003) and with DBS ON (p<0.001) compared to young controls (Table 6 and Fig 4). The main factor, vision, revealed that all body movements were larger with eyes closed than with eyes open both with DBS OFF (p<0.001) and with DBS ON (p<0.001). The main factor, adaptation, revealed that the PD subjects and young controls made marked reductions over time of body movements at all recorded sites both with DBS OFF (p<0.001) and DBS ON (p<0.001).

The significant interaction between Group x Vision revealed that knee movements were larger with DBS ON with eyes closed compared to all other conditions and young controls (p = 0.016). The significant interaction between Group x Adaptation revealed that head (p = 0.018) movements were proportionally reduced more than other positions but the hip (p = 0.041) movements reduced less with DBS OFF than in young controls. Moreover, the head, shoulders and hip (p≤0.044) movements were proportionally reduced less with DBS OFF than in young controls. The significant interaction between Vision x Adaptation revealed that the head, shoulders and hip (p≤0.035) movements were proportionally reduced more with eyes closed than with eyes open with DBS ON.

#### Effects of vision and adaptation in old vs young controls comparisons

The GLM ANOVA of the main group revealed that all body movements were larger in old controls (p≤0.006) compared to young controls (Table 6 and Fig 4). The main factor, vision, revealed that all body movements were larger with eyes closed than with eyes open (p≤0.006). The main factor, adaptation, revealed that old and young controls made marked reductions over time of body movements at all recorded sites (p<0.001).

The significant interaction between Vision x Adaptation revealed that the head and shoulder (p≤0.028) movements were proportionally reduced more with eyes closed than with eyes open during vibration.

### Movement amplitude changes to initial and continuous balance perturbations

#### PD with DBS OFF

With DBS OFF in the PD group, post hoc analyses showed that movement amplitude significantly increased at all positions in period 1 compared to quiet stance with eyes closed (p≤0.008) and eyes open (p≤ 0.004) (Table 7 and Fig 4). During the vibration period, from period 1 to period 4, the movement amplitudes decreased at the head, shoulders and hip (p≤0.010) with eyes open.

**Table 7:** Movement size changes between quiet stance and vibration period 1 and between vibration period 1 and period 4.

Movement size changes ^a^	Quiet stance vs Vibration Period 1		Vibration Period 1 vs Period 4	
	Eyes Closed	Eyes Open	Eyes Closed	Eyes Open
**PD OFF**				
Head	**0.004 (2.75)**	**0.004 (2.08)**	*0*.*031 (0*.*74)*	**0.004 (0.68)**
Shoulder	**0.004 (2.87)**	**0.002 (2.22)**	*0*.*047 (0*.*71)*	**0.010 (0.68)**
Hip	**0.008 (3.18)**	**0.002 (2.36)**	0.109 (0.78)	**0.006 (0.70)**
Knee	**0.004 (1.99)**	**0.002 (2.09)**	*0*.*031 (0*.*70)*	*0*.*049 (0*.*79)*
**PD ON**				
Head	**0.002 (3.59)**	**0.002 (2.59)**	*0*.*064 (0*.*82)*	1.000 (1.01)
Shoulder	**0.002 (3.76)**	**0.002 (2.73)**	**0.020 (0.83)**	0.846 (0.99)
Hip	**0.002 (3.34)**	**0.002 (2.73)**	**0.020 (0.86)**	1.000 (0.98)
Knee	**0.002 (2.96)**	**0.004 (2.19)**	**0.010 (0.79)**	0.570 (1.09)
**Old Controls**				
Head	**<0.001 (3.54)**	**<0.001 (2.79)**	**0.001 (0.71)**	*0*.*045 (0*.*84)*
Shoulder	**<0.001 (3.57)**	**<0.001 (3.15)**	**<0.001 (0.71)**	**0.020 (0.81)**
Hip	**<0.001 (3.34)**	**<0.001 (3.35)**	**0.001 (0.73)**	**0.003 (0.76)**
Knee	**<0.001 (3.14)**	**<0.001 (2.53)**	**<0.001 (0.69)**	**0.011 (0.77)**
**Young Controls**				
Head	**<0.001 (2.66)**	**<0.001 (2.41)**	**<0.001 (0.72)**	**<0.001 (0.73)**
Shoulder	**<0.001 (2.78)**	**<0.001 (2.58)**	**<0.001 (0.70)**	**<0.001 (0.72)**
Hip	**<0.001 (2.69)**	**<0.001 (2.48)**	**<0.001 (0.68)**	**<0.001 (0.68)**
Knee	**<0.001 (2.65)**	**<0.001 (2.57)**	**<0.001 (0.65)**	**<0.001 (0.59)**

^a^ The mean quotient value between quiet stance and period 1 and between period 1 and period 4 are presented within the parenthesis. A quotient value above 1.0 signifies increased movement sizes from the first to the second time period. The significant differences are marked in bold text and the trends in italic text.

#### PD with DBS ON

With DBS ON in the PD group, post hoc analyses showed that movement amplitude significantly increased at all positions in period 1 compared to quiet stance with eyes closed (p≤0.002) and eyes open (p≤ 0.004) (Table 7 and Fig 4). During the vibration period, from period 1 to period 4, the movement amplitudes decreased at the shoulders, hip and knees (p≤0.020) with eyes closed.

#### Old controls

In the old controls, post hoc analyses showed that movement amplitude increased at all body positions in period 1 compared to quiet stance with eyes closed (p<0.001) and eyes open (p<0.001) (Table 7 and Fig 4). During the vibration period, from period 1 to period 4, the movement amplitude decreased at all body positions with eyes closed (p≤0.001) and at the shoulders, hip and knees with eyes open (p≤0.020).

#### Young controls

In young controls, post hoc analyses showed that movement amplitude increased at all body positions in period 1 compared to quiet stance with eyes closed (p<0.001) and eyes open (p<0.001) (Table 7 and Fig 4). During the vibration period, from period 1 to period 4, the movement amplitude decreased at all body positions with eyes closed (p<0.001) and with eyes open (p<0.001).

## Discussion

The objective of this study was to investigate the effects of DBS in the STN on body position during quiet stance and balance perturbations. We found that patients with PD adopt a different body position with DBS ON vs. DBS OFF, to young and old controls, and with eyes open vs. eyes closed. The amplitude of movement during the vibration period decreased in controls at all measured sites with eyes open and closed (except at the head in old controls with eyes open) which contrasted the weaker effects of adaptation in patients with PD. Importantly, our results were independent to the effects of oral treatment.

We measured trunk flexion by comparing the angle between the upper body (head and shoulder) and ankle with the angle between the hip and ankle. An increase in trunk flexion was therefore indicated by an increased, positive, change of angle between the upper body and ankle in parallel to a smaller, or negative, change of angle between the hip and ankle. In patients with PD, we found a trunk flexion that varied in amplitude across participants. Trunk flexion was evident through an increased angle between the upper body (head and shoulder) and ankle, compared to the hip and ankle, in quiet standing. With eyes closed, the trunk flexion increased at the head and shoulder as shown by an increase in the forward position compared to the ankle. The first important finding is therefore that a visual reference (e.g., an image1.5m away) can partially abet trunk flexion in PD. However, the visual reference did not completely reverse trunk flexion consistent with an underlying central and peripheral pathology [35]. Corroborating a central and peripheral origin for the abnormal posture in PD, in a perceptual study of head position, patients with PD misperceived the upright position of their head [36] suggesting an underlying challenge integrating vestibular and visual cues and processing proprioceptive cervical information. These effects may be mediated at a subcortical or cortical level.

Treatment of PD with oral medication does not alter the degree of trunk flexion [37]. However, we found that DBS in the STN reduced trunk flexion in quiet stance, mainly with eyes closed, as shown in Fig 3. The effect of DBS was evidenced through local changes, with reduced flexion at the head, shoulder and knee and the hip was positioned further anteriorly. This repositioning was more consistent with the posture that controls adopted, although differences remained. With DBS OFF, the knees were further forward which ultimately reduced leaning forward of the shoulder as compared to DBS ON. The adopted posture with DBS OFF was also associated with an overall reduced movement amplitude. In contrast to the body position adopted by patients with PD, controls adopted an upright position. The posture adopted by patients with PD should not be viewed as being advantageous but may well be an inability to fully straighten the knee.

### Effects of PD vs controls on body position

Patients with PD adopted a posture with flexed knees though their shoulder and head were forward. The hips were positioned posteriorly as compared to the ankle. There was also greater forward position of the head and hip with DBS ON and DBS OFF as compared to young controls. These results suggest that although DBS can reduce the kyphotic body position in PD, it does not fully compensate for the effects, which is consistent with a previous study of quiet stance [38]. There is also an argument over the effectiveness of DBS in the STN vs. Globus Pallidus internus (GPi). In a case study of two patients with near crippling camptocormia (a trunk flexion exceeding 30° [39]), GPi DBS completely reversed the camptocormia and restored posture to normal [40]. Furthermore, in a small study of three patients with PD, two with bilateral DBS in the STN and one with bilateral GPi DBS, no or mild improvement of trunk flexion was seen in the two patients with DBS in the STN but a moderate improvement was seen in the patient with GPi DBS [41]. The results of the current study for DBS in the STN showed results that are more favorable (consistent with others, e.g., [4]) and could be a reflection of a longer interval between testing and DBS surgery. It should also be noted that in a systematic review by Chieng and colleagues [6], 68% of patients with PD saw an improvement to the degree of trunk flexion with DBS with an average reduction of 78.2% in flexion angle. Interestingly, a long-term benefit of DBS in the STN (five years post-operative) on trunk flexion has been suggested previously in a single patient case study [42] but another study with 25 patients points to benefits with shorter symptom duration [23]. That said, these inconsistent reports point to alternative pathogenic pathways. One study suggests that these differences attest to two subtypes: one group of patients with PD with basal ganglia predominant dysfunction who do respond to DBS and one group with greater myopathy who do not respond to DBS [43]. Other studies support the view that myopathy accounts for the trunk flexion [44, 45].

### Effects of adaptation on body position in PD

Calf vibration brought a change to the body position, with further leaning forward of the head and shoulder with DBS ON and OFF as compared to young and older controls. It may seem counter-intuitive to forward lean during balance perturbations but this change is consistent with the response of healthy controls. Calf vibration creates a proprioceptive illusion that the vibrated muscle is being stretched bringing about an increased firing from muscle spindles and a homonymous spinal reflex. Thus, perceived stretching of the calf muscle produces the perception of forward movement and the spinal reflex causes contraction of the calf muscles resulting in a posterior balance perturbation. When repeated, an adaptation is to lean the body further forward to prevent the apparent risk of falling backwards [13, 14, 33, 46]. The implication of this in our results is that patients with PD induce an adaptive change to their body position despite having a kyphotic posture already. The increased lean forward would bring about a possible risk of falling forward but in the event of a fall forward, the distance to the floor is narrowed and the center of mass is moved downward which decreases the overall length of the body (an indicative posture is shown in Fig 3).

The finding of further forward leaning could also be interpreted another way. The forward leaning shows that patients produce an inappropriate posture for the situation, which may suggest that the original posture in quiet stance, with increased trunk flexion, is treated by the CNS as the default position. This inappropriate adaptive adjustment implies poor interpretation of sensory information from muscle and joint receptors of the trunk and axial skeleton, and/or extrapyramidal effects on axial muscle tone. Interestingly, improving sensory feedback from cutaneous afferents has been shown to improve postural control in patients with PD [9] despite diminished joint proprioception [47]. Furthermore, advances in neuroanatomical methods, and in particular on the basal ganglia circuitry, has linked activity from the substantia nigra with the degree of muscle tone as part of the extrapyramidal system [48] suggesting a complex central and peripheral involvement in kyphotic postures.

The standing posture in PD also plays an important role in gait. In studies of gait in PD, the standing posture has been found to determine freezing of gait and step length [49]. It is therefore reasonable to suggest that an improved posture with DBS in the STN in patients with reduced trunk flexion would have positive effects on gait.

### Effects of adaptation on movement amplitude in PD

During balance perturbations, patients with PD and controls demonstrated adaptation as movement amplitude decreased with calf muscle vibration. When balance is perturbed, the brain detects the imbalance and makes changes to body posture and generates predictive muscle responses to reduce the imbalance when the perturbation is repeated [30, 50–52]. This adaptive response is mediated initially by cortical mechanisms and thereafter by subcortical mechanisms [53, 54]. However, there were differences in the adaptive response between groups, which was influenced by having eyes open or eyes closed and in patients with PD, regardless of whether DBS was ON or OFF. Despite the adaptation, body movement was larger in patients with PD as compared to young controls, but not old controls, across all periods. There was also a greater reduction of body movement with eyes closed as compared to eyes open, which attests to greater levels of imbalance with eyes closed, over eyes open, in vibration Period 1 and adaptive mechanisms that are independent to visual feedback.

In patients with PD, with DBS OFF, movement amplitudes decreased at the head, shoulder and hip over time with adaptation with eyes open but not eyes closed. With DBS ON, movement amplitudes decreased at the shoulder, hip and knee with eyes closed but not with eyes open. This contrasts old and young controls who produced a reduction of movement at all sites, except at the head in old controls with eyes open. Overall, shoulder movement reduced the least in patients with PD as compared to old controls, consistent with increased shoulder rigidity with trunk flexion [55]. These findings suggest that alterations of posture, associated with greater forward leaning as calf vibration was repeated, are independent to movement amplitude changes. It is interesting to note that patients with PD experience greater reduction of trunk flexion with DBS in the STN if there is greater structural connectivity between the motor planning areas (right lateral premotor cortex and right supplementary motor area) and volume of tissue in the STN activated by DBS [4]. These cortical motor planning regions would be activated during the vibration task to generate adaptive responses. Therefore, the reduced levels of adaptation for movement amplitude in patients with PD could be because the motor planning regions are either disconnected (DBS OFF) or occupied with the regulation of trunk position (DBS ON).

### Effects of vision on body position and movement amplitude in PD

An interesting finding was the effect of PD and DBS on the position of the knee. However, knee position in patients with PD was also affected by vision, where the knees were further flexed with eyes closed. There was evidence that the degree of trunk flexion alters the position of the knee. In line with this, bracing the thorax and lumbar regions in patients with PD to upright causes the knee and hip positions to align to the corrected position of the trunk (i.e., there is greater extension of the knee) [56]. Furthermore, greater flexion of the knee could reflect joint stiffening through co-contraction [57] meaning that an increased trunk flexion has widespread effects. However, the overall influence of vision on body position was less marked than we have previously found in experimentally intoxication participants [33]. Observing an image ahead provides a vertical and horizontal reference, allowing participants to perceive the position of the head during quiet stance and during balance perturbations. This sensory information is transmitted to the central nervous system to be integrated with vestibular and somatosensory inputs. Here, impaired integration of visual information could alter the contribution of the visual frame and explain the lower effectiveness of vision in patients with PD. Concordant with this explanation, a centrally-mediated deficit in processing visual information has been found in patients with PD during Achilles tendon vibration [58].

### Study limitations

The study has limitations. Firstly, the sample size of the PD population was relatively small. However, we employed strict selection criteria to provide a homogenous population and therefore our results are likely to translate to a larger population of patients with PD with DBS in the STN. A further limitation is the absence of a group of patients with PD who have not yet received DBS in the STN.

## Conclusions

To conclude, body position is altered in patients with PD with greater trunk flexion and knee flexion as compared to controls. However, here we show that DBS in the STN can significantly improve body position although the effects are not completely reversed. Body position in PD was also affected by vision and adaptation to vibration.

## Supporting information

S1 Dataset (XLSX)
